# Atomic Force Microscopy: A Powerful Tool to Address Scaffold Design in Tissue Engineering

**DOI:** 10.3390/jfb8010007

**Published:** 2017-02-13

**Authors:** Marica Marrese, Vincenzo Guarino, Luigi Ambrosio

**Affiliations:** 1 Faculty of Sciences, Biophotonics and Medical Imaging group and Laser Lab, VU University Amsterdam, De Boelelaan 1081 HV Amsterdam, The Netherlands; m.marrese@vu.nl; 2 Institute of Polymers, Composites and Biomaterials, National Research Council of Italy, V.le Kennedy 54, Pad 20, Mostra d’Oltremare, Naples 80125, Italy; ambrosio@unina.it

**Keywords:** Atomic Force Microscopy (AFM), conductive AFM, thermal analysis, phase imaging, nano-indentation, force spectroscopy, electrospun fibres, films, micro/nano particles

## Abstract

Functional polymers currently represent a basic component of a large range of biological and biomedical applications including molecular release, tissue engineering, bio-sensing and medical imaging. Advancements in these fields are driven by the use of a wide set of biodegradable polymers with controlled physical and bio-interactive properties. In this context, microscopy techniques such as Atomic Force Microscopy (AFM) are emerging as fundamental tools to deeply investigate morphology and structural properties at micro and sub-micrometric scale, in order to evaluate the in time relationship between physicochemical properties of biomaterials and biological response. In particular, AFM is not only a mere tool for screening surface topography, but may offer a significant contribution to understand surface and interface properties, thus concurring to the optimization of biomaterials performance, processes, physical and chemical properties at the micro and nanoscale. This is possible by capitalizing the recent discoveries in nanotechnologies applied to soft matter such as atomic force spectroscopy to measure surface forces through force curves. By tip-sample local interactions, several information can be collected such as elasticity, viscoelasticity, surface charge densities and wettability. This paper overviews recent developments in AFM technology and imaging techniques by remarking differences in operational modes, the implementation of advanced tools and their current application in biomaterials science, in terms of characterization of polymeric devices in different forms (i.e., fibres, films or particles).

## 1. Background: Advantages and Limitations

Microscopy is the most important instrument for morphological studies in biomedical field. Traditionally, optical microscopy based on the transmission of light supported by different glasses and lenses has been largely used for the investigation of morphological properties at micrometric size scale.

During the last century, new discoveries about interaction mechanisms of electrons–diffraction or transmission ones–with the matter, have promoted the development of scanning and transmission electron microscopy (SEM and TEM) able to drastically increase the resolution quality of morphological investigation, thus providing further morpho-structural details at sub-micrometric and nanometric size scale [1]. 

In the last decades, microscopy technologies based on innovative lenses, photons, or electrons, have been investigated to explore material surface, in order to collect new morphological, structural and mechanical information from biological systems. About 20 years ago, Binning, Quate and Gerber [2] developed a new type of microscope able to measure forces on an atomic scale on graphite samples [3,4], thus providing a first concrete idea of current scanning tunneling microscopy (STM) and Atomic Force Microscopy (AFM) [5,6]. Hence, atomic force microscope is become one of most widespread technology based on the use of micro- and/or nano-structured probes for scanning materials surfaces at sub-nanometric and atomic resolution, able to investigate organic and biological properties from microns down to molecular scale [7]. 

More interestingly, continuous improvements in AFM technology now allow punctually evaluating interface properties, by measuring the interaction forces of the probe with extended surface domains down to single molecules. This is really attracting worldwide the interest of scientists, mainly due to the unique opportunity to perform a broad range of analyses (i.e., mechanical, chemical and functional properties) under different conditions (i.e., liquid microenvironment, microgravity, low temperature [8]) for potential application in different biomedical fields.

The main advantages of recent improvements in AFM technology undoubtedly concern their use in tissue engineering (TE) and regenerative medicine (RM). The first scope of TE is to assemble functional constructs or scaffolds able to restore, maintain, and improve injured or damaged tissues. In this context, it is crucial to investigate specific interactions between single cells and extracellular matrix, into micro/nanostructured scaffolds made of natural or synthetic polymers, under specific stimuli exerted by surrounding biological environment at micro and nanometric scale [9].

In this context, AFM technique is rapidly emerging as a gold standard technique to collect more detailed information about surface and interface properties respect to other microscopy techniques. This is mainly due to the high accuracy to measure adhesive forces in different materials sensitive regime [10,11,12,13,14] and the translation of mechanical theories previously applied to hard materials just for macroscopic tests (i.e., metals [15]) to soft matter like tissues. 

Moreover, AFM is gaining an added value as useful tool for scaffold design, giving the opportunity to perform a wide range of sample testing on biopolymers under physiological conditions, without any complex preparation procedures (i.e., sample fixation, cryo-preparation methods [16,17]). 

Accordingly, this review will focus upon topographical, structural and chemical applications of AFM in tissue engineering by providing a concise balance of their potential advantages and limitations. After a brief analysis of AFM working principle and their application modes (i.e., contact, non-contact and tapping modes), we underline the main features of AFM analyses as high versatile morphological tool to scan a large variety of bioinspired surfaces, i.e., synthetic walls of scaffolds [18], biological systems [19], nanostructures [20] and biopolymers [21], with a planar resolution ranging from nanometric down to atomic scale. Secondly, we deeply analyse the use of AFM as force spectroscopy technique [22] for a quantitative study of average or local adhesion or mechanical behaviour ascribable to inter and intramolecular forces occurring in biological systems [23] single cells, or polymer constructs. We conclude this review by examining recent innovation in AFM technologies, by discovering advanced tools such as Conductive and Thermal analyses which are forcefully emerging for their potential use in biomedical field.

## 2. Working Principles and Basic Tools

**Figure 1:**
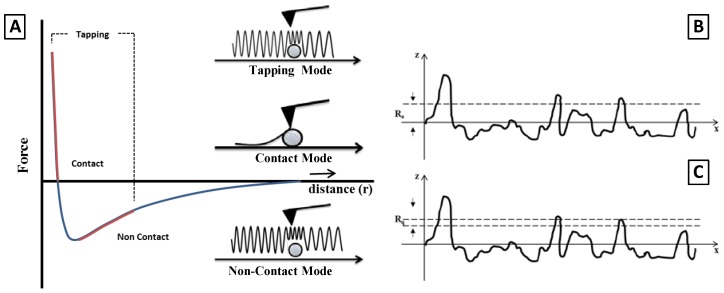
(**A**) Tip-sample separation curve illustrating the main interaction during Atomic force microscopy (AFM) scanning. The AFM imaging modes for each regime are also shown. Graphical definition of (**B**) Average roughness $R_{a}$ and (**C**) Root Mean Square Roughness (RMS) Roughness ($R_{q}$ ). This figure is freely inspired by [25].

In tissue engineering, atomic force microscopy is commonly used for the topological investigation of surfaces of biomaterials—also in the presence of adhered cells—with atomic resolution. In this case, the operative approach is well-known, so replying consolidated procedures just used in material science to study surface topography of traditional classes of materials (i.e., polymers, ceramic, metals) [24]. The working principle is based on the use of a micrometric tip, properly fixed to a cantilever, which is brought in the proximity of a sample surface (Figure 1) [25]. The interatomic interaction among atoms composing tip and surface is evaluated. While the tip scans the sample surface, tip oscillations occur as a consequence of changes in the surface topography, thus recording interatomic potential variations. In particular, by monitoring the deflection of the cantilever, the morphological features of the surface can be observed, case by case, properly setting operational conditions.

A proper classification of the AFM operation modes can be done as a function of force interactions between tip and surface (Table 1). In particular, AFM works in “contact mode” in presence of constantly repulsive forces or in “non-contact mode” in presence of attractive forces onto the tip. Lastly, AFM may work in tapping mode in presence of both attractive and repulsive force [24]. For all the operation modes, the images may be reconstructed by recording all interatomic interactions occurring at the end of the tip during the cantilever scanning onto the sample surface [26].

### 2.1. Contact, Non-Contact and Tapping Mode 

The contact mode is the firstly discovered imaging mode and occurs when the tip is steadily in contact with the sample surface. Meanwhile, a feedback system is generally used to keep the cantilever deflection constant during scanning [5]. This operational mode is the most suitable for flat and rigid surfaces such as crystal, hard polymers and tissue, enabling the highest resolution level. In this mode, it is possible to capture image artefacts related to a not-flat surface or to a mechanical drift derived from the scanner motion. These artefacts can dominate the topography image over the real morphological features. To avoid these artefacts and to measure how well the desired deflection set point is maintained constant by the feedback system, the error mode can be used [27]. In particular in order to display a true “height image” a negative feedback loop is used. When this feedback system does not work properly, small cantilever deflections may occur, which will be reported as an error signal onto the “deflection image.” Height image is generally used for studying the quantitative height topography, surface roughness, and thickness of biological layers, while the deflection image is used to reveal finest surface details, working in response to higher frequencies [28]. To date, contact mode analyses may be realized also in aqueous medium in order to minimize interatomic forces occurring at the tip/sample interface, ascribable to the surrounding environment (i.e., air flow, etc.). The ability to conduct experiments in aqueous environment has given a new insight for the study of biological materials, in their physiological conditions as confirmed by recent studies of liquid- environmental AFM imaging [29,30]. In this case, the surface contribution is very low as well as the capillary forces, as a consequence of the water vapour condensation onto the surface of the AFM tip, during the residence in air.

However, large AFM tip-sample interactions, commonly measured in contact mode, are not suitable for detecting the presence of single atoms, small molecules or defects along the surface of soft materials. Moreover, the direct contact of the tip onto soft materials (i.e., cells, biological matter) may easily alter the surface with undesired sample damage. This may be prevented by operating in non-contact mode, it means by forcing the cantilever oscillation at resonance frequency. In this case, the oscillating probe is generally influenced by the close surface, so producing a frequency shift in the resonant frequency, due to van der Waals attractive forces. Hence, the signals recorded in non-contact mode are related to the variation between cantilever resonance frequency and free oscillation of the system, giving a preliminary estimation of atomic tip-sample interaction forces intensities. However, non-contact-AFM image may be determined by frequency variations related to fluctuations in frequency shift as direct signal (constant height image) or as a feedback-loop signal (constant frequency shift image). As a consequence, resonance frequency and oscillation amplitude both concur to the surface image formation. Moreover, in order to minimize the attractive forces onto the tip close to the surface, the spring constant used in non-contact mode is greater than in contact mode. Major drawbacks of non-contact mode concern low lateral resolution and constrained use of the tip in air environment [24].

Lastly, in the case of tapping or intermittent mode, probe excitation externally occurs and the amplitude and cantilever phase may be monitored in the proximity of the resonance frequency. Respect to the other operation modes, tapping mode has been employed successfully for the topographic characterization of cell loaded surfaces, due to the application of lower forces and high resolution imaging [28]. The general working principle is similar to those used in non-contact mode. However, the tip contact at the sample surface occurs very falsely during each oscillation cycle. Due to the energy loss caused by single intermittent contact, the vibration amplitude drastically changes as a function of the peculiar topography of the sample surface. In particular, when the tip encounters a protrusion on the sample surface, the amplitude of cantilever oscillation decreases due to a reduction of the vibrating space. Contrariwise, the vibration amplitude increases in the presence of concave surfaces. As a consequence, the tip is interested by both attractive and repulsive force during each oscillation in tapping mode [3], but tip/sample interactions are commonly smaller in comparison with contact mode, so improving higher vertical and lateral resolution.

More recently, phase imaging working in tapping mode is emerging as a suitable tool to collect information about surface properties, not detectable by other AFM modes. This approach could be really interesting in tissue engineering to collect significant information about viscoelastic properties of biomaterials, by monitoring the phase shift between cantilever oscillations and its driving signal [31]. In particular, the identification of physical domains with different composition and material properties is possible by correlating the phase delay to the energy loss during the tip–sample interaction. 

**Figure 2:**
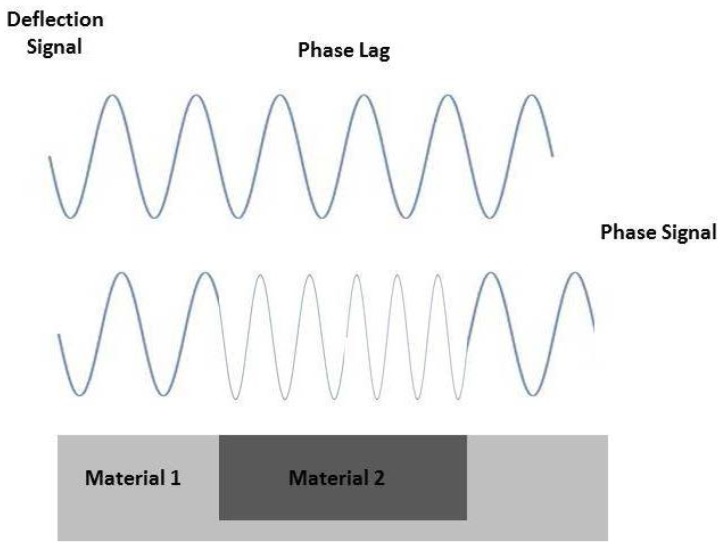
Phase signals in the AFM tapping mode arising from phase shift in the tapping signal, depending on peculiar material properties of the sample surface.

The phase imaging tool also allows recognizing regions with different elasticity (Figure 2) [24,32] or other structural heterogeneities, such as crystalline/amorphous composition of polymers, by recording offsets and phase angles of input signal variations respect to those of the oscillating cantilever. In particular, the visualization of amorphous and crystalline components in polymers is extremely interesting, since it is able to highlight edges and it provides fine observation of peculiar structures, which are often obscured by a rough topography investigation [33].

### 2.2. Roughness Data

AFM has been traditionally evaluated as a powerful technique to perform an accurate measurement of surface roughness as well as to investigate failure mechanisms of polymer scaffolds [32,33,34,35]. The understanding surface properties at sub-angstrom size scale are becoming tremendously relevant to explore the main features of scaffold at nanoscale. In order to gain a deep understanding of the interaction between cells and their biological surrounding, a broad class of materials and biomaterials have been used to fabricate porous scaffolds in tissue engineering. To date, it is well recognized that adhesion as well as spreading and proliferation of a cell lines is significantly influenced by the physicochemical properties of the surface [36,37]. In this context, wettability, roughness and topography play a crucial role on the basic mechanisms of cell–biomaterial interactions. For this purpose, high lateral and vertical resolution of AFM analyses [34] make them is extremely more sensitive than traditional profilometer techniques to measure surface roughness, However, roughness value depends on several scanning parameters which are strictly influenced by height variation of sample profile, thus resulting not suitable to investigate the surface of macroporous scaffolds, i.e., some hundreds of micrometres as diameters, commonly used for cell hosting in tissue engineering.

However, roughness measurement from raw height data obtained via AFM may offer extensive information on the modelling of cell materials interactions. A broad range of surface roughness definition are being given in the metrology community and most of them are based on a statistical representation of the vertical height deviations, called peaks, from a mean line taken as a reference. A considerable number of definition have been proposed to describe the roughness, in particular Wennerberg and Albraktsson, in 2000 [38], proposed the use of 2D height parameters: Average roughness $\left(R_{a}\right)$ and Root Mean Square Roughness $\left(R_{q}\right)$ as the most suitable parameters for roughness characterization of biomedical scaffolds (Figure 1B,C). Essentially, the roughness of any surface is quantified by measuring vertical deviations of the measured surface in comparison with a reference profile. In particular, $R_{a}$, by far the most common, is mathematically given by the Equation (1):

(1) $$R_{a}=\frac{1}{N}\;\overset{N}{\underset{J=1}{\sum }}\left|Z_{J}\right|$$

where N represents the total data points involved in the measurement, while $Z_{J}$ is the vertical deviations measured from the average height of the surface.

In contrast, RMS roughness value ($R_{q}$) is expressed as root-mean-square of the average height of the surface. The definition of RMS roughness is more sensitive to small changes in the raw profile of the sample due to a misinterpretation of the data set or to the equipment that are used to perform such experiments. For this reason, this parameter is also more sensitive to large deviation from the mean line as opposed to $R_{a}$ [38]. The mathematical definition of $R_{q}$ is as follows:

(2) $$R_{q}=\sqrt{\frac{\sum {\left(Z_{J}\right)}^{2}}{N}}$$

where N is referred to the data points while $Z_{J}$ is the vertical deviation of j-th point with respect to the mean line. In Figure 1B,C a graphic explanation of the two parameters $R_{a}$ and $R_{q}$ is reported. Besides, the roughness values are sensitive to the pixel resolution and size. Hence, the use of implicit filter—according to the ASME and ISO metrological standards—to correct images for tilt and bow (i.e., plane fitting) can dramatically affect the roughness calculation. Thus, a correct sizing of surface to scan is recommended to minimize unexpected artefacts due to filtering effects [39]. A similar approach has been adopted by Weng et al., by using $R_{a}$ and $R_{q}$ parameters to discriminate cancerous and benign breast epithelial cells as a function of the roughness data [38].

### 2.3. Micro\Nano Indentation

One of the most crucial features of AFM in nanoscience is referred to its capability to characterize the mechanical properties of scaffolds, polymers, proteins, cells and bacteria [9]. In particular, the ability to quantify viscoelasticity of any natural and synthetic polymers is providing new important information for biomedical studies addressed to explore the relationships between the mechanical behaviour and physical and/or chemical properties. To date, AFM offers the more efficient non-destructive way to quantitatively estimate multiple properties (i.e., elasticity, viscoelasticity, plasticity, hysteresis, hardness, compliance, adhesion force and stiffness), also providing an immediate overlap with a broad set of topographical information as a function of the specific operational mode used [9]. In this context, indentation represents one of most interesting method based on AFM to investigate and locally measure mechanical properties. It is possible to distinguish between micro and nano indentation according to the indenter dimensions, as the size of the spherical tip varies from tens of nanometre until several micrometres. In such applications, the tip is pushed further towards the sample until a certain depth (penetration depth). The cantilever bending is monitored by the AFM photodetector system, and thus, the mechanical compliance of the tip-sample contact can be extrapolated. Cantilever deflection and tip penetration into the sample are usually modelled with Hertzian contact mechanics. This theory is based on geometrical constraints of two spherical shapes in contact under a perpendicular load. Linear elastic deformation of both shapes is assumed with a quadratic pressure distribution along the area of contact. The model assumes that the surfaces in contact are continuous, smooth and non-conforming; the strains implied in the indentation are small, and both the indenter and the sample can be described as elastic half-space. In the Hertzian theory, surfaces must be adhesionless and frictionless while stiffness homogeneously distributed along the sample. Moreover, the contact geometry is assumed to be axisymmetric, smooth and continuous, while, the tip has to be spherical, undeformable and infinitely stiff compared to the sample. 

The first elastic model of indentation, developed by Hertz [40,41], is currently used in the nano range, despite the use of some restrictions. Wide types of indenters are being used for indentation techniques. The most commonly used probe is characterized by a spherical tip for their easy applicability, but sharp indenters such as pyramidal or Berkivich tips are generally preferred for the investigation of biological materials.

**Figure 3:**
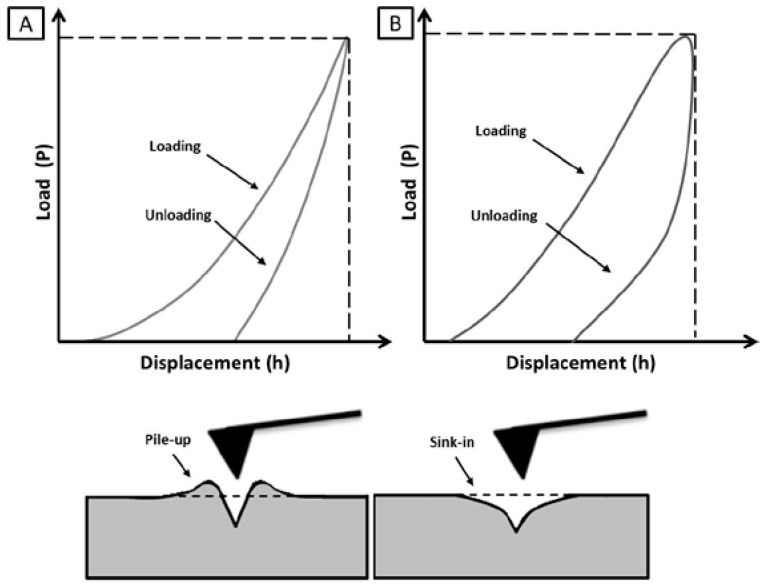
Schematic illustration of load vs. displacement curve on hard elastoplastic material (**A**) and distorted curve due to viscoelastic and plastic response on soft materials (**B**), derived from atomic force spectroscopy and indentation curve (first line); pile-up and sink-in phenomena after unloading using sharp indentation (second line). This figure is freely inspired by [46].

In the last three decades, Hertz theory been extended by Sneddon to different indenter geometries [41]. Doerner and Nix (1986) [42] have used flat punch geometry to evaluate the contact area between the tip and sample, while extrapolating elastic modulus from force–deformation curves obtained by the Hertzian theory. Most recently, Oliver and Pharr (O&P) have redefined this theory in order to take in account the changing in the contact area at different indentation depth in the unloading curve (Figure 3, first line) [43]. Their approach was aimed at measuring elastic and plastic deformation with the assumption of linear elastic deformation along the upper part of the unloading curve. In this case, Young’s Modulus was derived directly from the unloading slope of indentation load/displacement curve, defined as ratio between stress/strain [44]. Hence, indentation model has been routinely used to describe linear elastic and plastic properties of different 2D materials, i.e., thin films, modified interfaces, biological tissues and surfaces of porous scaffolds [45].

During the indentation test, the presence of capillary and adhesive forces changes the contact profile, also modifying local force acting between the tip and the sample. Several models such as JKR theory [47] or DMT model [48] have been implemented to take into account adhesion forces exerted during indention tests. JKR is mainly referred to the indentation on soft materials whereas DMT for indentations on stiff ones. However, their applicability has been recently extended to all materials by the introduction of Tabor coefficients—defined as the ratio between the depth of penetration and the equilibrium separation between two surfaces in contact [49].

During indentation tests, another critical point concerns the deformation of sample around the contact point, i.e., pile-up or sink-in (Figure 3, second line). The phenomena of pile-up and sink-in are both associated with nano-indentation in case of elastic-plastic material. In particular the pile up leads to contact areas that are greater than the cross sectional area of the indenter at given depth while the sink in leads to contact area than are less than the cross sectional area. These deformations have been found to have large effects on the measurements of indentation moduli since these phenomena can significantly influence the contact area and consequentially that can lead to inaccurate measurements of the indented material. Highest pile-up is usually detected on the assumption of elastic-plastic materials, while, highest sink-in occurs in the case of strain hardening behaviour occurs (i.e., nanocrystalline materials). It has been demonstrated that different materials can be differently deformed in the proximity of the contact point. Therefore, these deformations cannot be correlated to the applied load but only to the intrinsic material properties [50]. 

For this purpose, Ah-Young Jee and Minyung Le [51] have investigated the local deformation around the contact point in the case of different polymeric membranes by using both force–displacement curve and area–depth images. Compared with conventional indentation, AFM analysis is able to collect information about contact area and depth penetration simultaneously from imaging data and/or load–indentation curve. In this case, O&P theory may be applied to extrapolate the mechanical behaviour of polymers from the load/displacement curve, thus obtaining similar Young modulus values respect to concurrent methods based on imaging data. 

## 3. Major Applications in Scaffolds Design

The investigation of cell material interactions is pivotal to collect relevant information about the effect of structure and composition at atomic and macromolecular levels of bioinspired scaffolds on biological events. So, the evaluation of surface properties may be suitable for this scope. Optical microscopy has been traditionally used to in vitro evaluate multi-dimensional features of 3D scaffolds and their multi-parametric dependence by intrinsic and environmental factors. However, microscopic analyses show relevant drawbacks in terms of accuracy due to blurring effects at greater depths light scattering, which limit the capability to collect morphological information in the presence of sub-micrometric domains or crystals, able to influence local interactions at the molecular level.

**Figure 4:**
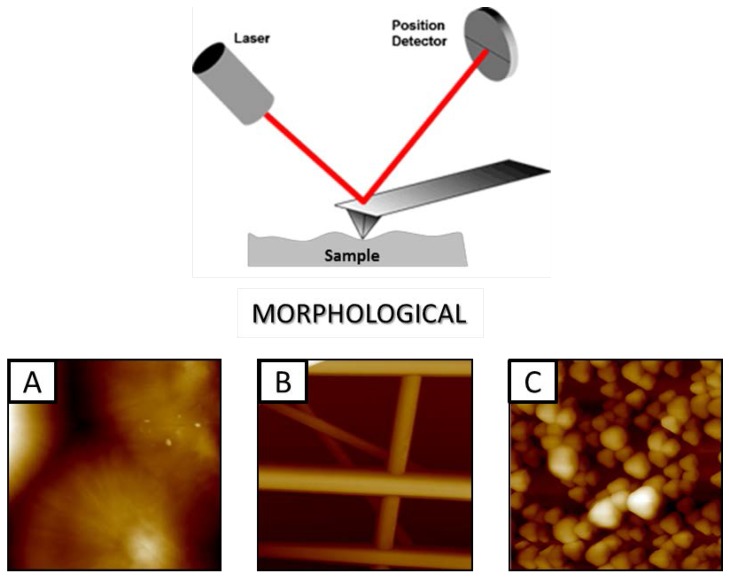
AFM morphological images of (**A**) solvent casting poly(ε-caprolactone) (PCL) films (scan area 50 × 50 µm^2^) [53]: (**B**) PCL-Gel electrospun membrane (scan area 20 × 20 µm^2^) [54]; (**C**) PLGA electrosprayed microparticle (scan area 20 × 20 µm^2^).

The discovery of microscopy based on electron emitted sources rapidly allowed overcoming main limitations of optical microscopy, offering a more efficient tool to investigate biological tissues or cellularized materials on the micro- and nanoscale levels. By the use of environmental chambers with temperature and humidity controls, it is possible to reproduce similar conditions to the natural state of biological samples without freezing of samples. However, there are still some limitations concerning the presence of artefacts related to the effect of applied environmental conditions, which may alter the effective structure of polymer scaffolds. More recently, AFM is emerging as an alternative technique with relevant impact on the in vitro investigation of biodegradable materials fabricated in different forms, i.e., films, fibres, or nanoparticles for tissue engineering and drug delivery applications (Figure 4), able to gradually resorb by forming an ex novo extra cellular matrix with predefined structural/mechanical properties similarly to native tissues [52].

For instance, in hard tissues regeneration, it is extremely important the use of mechanically stable scaffolds with strength and stiffness able to support biological loads during the first steps of formation until the complete tissue maturation. In this regard, AFM supported by recent advanced tools may be suitable to investigate surface properties of porous scaffolds, thus collecting detailed information on morphological, chemical, physical, mechanical properties useful to more deeply understand biological response of cells during in vitro culture. In this section, we introduce an overview of major applications of AFM technology to support scaffold design for in vitro cell response screening. 

### 3.1. Investigation of Structural Properties

When cells adhere to surface of a scaffold, a sequence of physico-chemical reactions will happen between cells and the scaffold. In the last years, several studies have been focused on the biomaterials processing in order to properly modify surface properties to promote and controlling key factors which govern cell response. For instance, it is universally recognized that characteristic size of fibres into nanometre range, due to the higher extend of surface respect to volume, may drastically increase biological response, by exerting novel and unique physical, mechanical and electrical properties [55] able to improve cell interactions at the tissue interface. More interestingly, AFM also offers the opportunity to investigate the effect of morphological and biochemical signals provided by multifunctional scaffolds [56,57]. For instance, Li et al. have used this approach to evaluate the contribution of phase morphology along the fibre surface on mechanical behaviour of electrospun silk fibres [58]. Accordingly, Zhang and co-workers [59] have explored surface morphology of porous nanofibres made of poly(ε-caprolactone) (PCL) and Gelatin. In this case, phase imaging AFM has been successfully used to explore phase separation mechanisms occurring during solvent extraction, giving the opportunity to regulate process conditions in order to obtain controlled pore sizes and preferential pore orientation along the fibre axis. 

Moreover, AFM analysis may be successfully used to identify the spatial organization of different polymer phases in the case of polymer blends with peculiar composition, by providing a mapping of different domains, as a function of their intrinsic viscoelastic properties. In this case, AFM phase images show surface stiffness variations ascribable to changes in the Young’s elastic moduli [58]: stiffer regions refer to brighter area into the map, related to positive phase shifts, while soften regions to darker ones, thus giving the chance to detect and quantify functional properties in selected area of multicomponent systems [58].

Besides, AFM analyses may significantly contribute to collect relevant information about the mechanical response of scaffolds. It is well-known that mechanical properties of the extracellular matrix (i.e., rigidity) are fundamental to influence cell fate during in vitro and/or in vivo practices. In this context, several studies demonstrated that bulk properties are not completely deputed to define the effective mechanical behaviour of scaffolds, but surfaces and interfaces also play a relevant role on local response. This aspect is mostly relevant in the case of electrospun scaffolds. Indeed, the peculiar topological and morphological features of the fibre network, i.e., fibre diameter, size distribution and roughness–drastically influence the mechanical boundary conditions at the nodes of the fibre network, thus particularizing the ultimate mechanical behaviour of the fibrous scaffold. 

Besides, electrospun fibre networks present highly complex structural organization which makes really challenging the study of tension and bending moduli variation effects on the final response in biological context [59]. For instance, under simulated physiological conditions, fibres are subjected to peculiar stresses able to generate permanent deformation until the mechanical failure, strictly related to the properties of the local biological niche. Therefore, the prediction of local mechanical forces may contribute to understand the crosstalking mechanisms—cell-to-cell and cell-to-material—at the nanoscale level, and their effect on in vitro response. Moreover, the opportunity to evaluate the mechanical response along the single fibre by assessing micro-mechanical bending tests via AFM allows for the determination of bending and shear moduli [60] of single fibres or their portions.

In this context, the use of surface modification treatments able to improve morphological integrity of fibres and mechanical response can be also evaluated by properly using atomic force microscopy tools. Recently, Pappa et al. [61] have investigated cell attachment and growth onto poly-ε-caprolactone (PCL) electrospun nanofibrous scaffolds treated by oxygen plasma, suitable for coatings of cardiovascular implants. In this case, AFM was used to determine topographical features and to evaluate variations in surface roughness due to the proposed treatment. Similarly, Golomb and Sahin, in 2013 [60] have performed mechanical tests on plasma treated silk electrospun fibres by obtaining a mapping of elastic-modulus and stiffness determined by torsional harmonic AFM. In this case, the use of a clamped-end model—based on the assumption of displacements and bending of a fibre restricted at nodes—have accurately confirmed measured experimental data of stiffness variations. Accordingly, Chlanda et al. [62] have proposed a comparative mechanical study among four different polymer based electrospun fibres by Peak Force Quantitative Nano Mechanics (PFQNM) atomic force microscopy, with standard and modified scanning probes. PFQNM is a recently developed imaging technique, based on nanoscale probing able to measure mechanical properties of constituent materials—including Young’s modulus, adhesion, loss modulus—with highest spatial resolution, by tracing force-distance curves after DMT (Derjagin, Muller, Toropov) [48]. Results clearly indicate that modified probes produce high quality images respect to standard tips, providing more detailed data on nano-mechanical response in comparison with traditional approaches [62].

Some modifications are generally required to investigate structural properties in the case of polymer films, due to relevant difference in mechanical properties respect to those of bulk systems made of the same constituents. In this case, strong efforts have been spent to implement non-destructive or, at least, less invasive techniques able to investigate blends or copolymers by probing ultrathin films and layers, without any reduction of the imaging resolution, due to the presence of structural inhomogeneity [63]. In this context, AFM presents the unique capability of exerting ultralow forces on sample surface by local single-point mechanical probing. This allows for testing compliant materials such as the most part of biopolymers and nanocomposites for TE use, in the form of thin and ultra-thin films on stiffer substrates, also quantifying changes in composition by phase imaging [64]. In particular, this technique allows detecting contrast variations, due to viscous energy dissipation in the presence of different viscoelastic domains along the surface. It is well-known that polymers show a peculiar viscoelastic behaviour described by the strain stress response lags and quantitatively detected by phase angle as a function of the specific material. Scott et al., [65] demonstrated a strong dependence of phase angle contrast to viscoelastic properties at high vibration amplitude and low tip-to-sample distance. In particular, lower viscoelastic properties can be detected for lower phase angles and vice versa, thus simultaneously providing topographic and chemical information of clean or cell loaded surfaces [65].

Several works have successfully used AFM working in both contact and tapping mode to detect some differences in films morphology. For instance, Cardenas et al. [66] have used AFM to characterize morphological properties of chitosan composite films to investigate the influence of different solvents (i.e., acetic or lactic acid) and additives on surface roughness. By the evaluation of root mean square roughness, they verified that chitosan lactate films present less roughness than the chitosan acetate ones, also resulting more homogeneous in the presence of the different additives. More recently, Stan and co-workers [67] reported the AFM investigation of polymer composites based on poly (4-styrenesulfonic acid) (PSSA) and polyaniline (PANI) with different molar ratios, evidencing an increase of surface irregularities as the PSSA percent increases. Similar effects have been recognized by Gambardella et al. investigating the morphology and the conductive properties of Hydroxyapatite films enriched with magnetite. In this case, roughness affects bacterial adhesion mechanisms triggered by porous/rough surfaces properties, ultimately inhibiting cells adhesion [68]. 

Lastly, AFM can be used as successful tool to investigate polymeric devices in the form of micro- and nanoparticles for drug delivery, tissue engineering and bioengineering applications. In the last years, several scaffolds loaded with nanoparticles have been designed for the site-specific delivery of drugs and biomacromolecules. In this context, targeting properties of nanoparticles may be drastically influenced by specific surface properties, i.e., local charge, surface modification and hydrophobicity. In vivo response of nanoparticles may be further influenced by morphological characteristics, i.e., size, shape—which may drastically affect target and route of drug administration. In this context, AFM is emerging as a reliable methodology to investigate microparticles from 1 nm to 5 µm in in size by single scan. Different AFM analyses can be performed as a function of the specific preparation of the substrates; for instance, particles can be rigidly attached to a solid structure or dispersed in liquid. AFM tip modification or nanoparticle manipulation may be also useful to study nanoparticles interactions by AFM probing [69,70,71]. 

In the case of flat substrates, size of isolated nanoparticles can be easily, but accurately, determined as measure of nanoparticle image height [70] without any tip-sample convolution effects. However, in the case of rough substrates, size analysis cannot be easily performed but particle analysis methods have to be, case by case, implemented as a function of specific boundary conditions. More frequent problems concern the influence of particle agglomeration and self-ordering during AFM scan onto the surface. In this regard, Klapetek and co-workers [72] have assessed a statistic model to evaluate the influence of substrate roughness and particle agglomeration during of nanoparticles size measurements. In particular, they propose the use of different processing algorithms to estimate statistical uncertainties derived from AFM analyses under non-ideal conditions in order to obtain quantitative information about nanoparticle morphological features (i.e., average size and distribution). By the nanoparticles attachment to the scanning probe, it is possible to measure single nanoparticles interactions or their adhesion force onto the substrate [73]. In this regard, Vakarelski et al. have paved the way for the development of functionalized AFM tips with nanoparticles in order to indent and penetrate the membranes of living cells [74].

### 3.2. Investigation of Chemical Composition/Functionalization 

Currently, AFM is considered the best tool for surface characterization, suitable to investigate morphological properties of polymers and biomaterials, by outputting high-resolution images able to reproduce micro- and nanotextured surfaces of 3D scaffolds for biomedical applications. Moreover, it allows distinguishing materials phases by using phase contrast modes, in the case of micro and nanocomposite systems, thus offering interesting functions for understanding local in vitro interaction mechanisms at the interface with cells. Recently, an increasing number of studies suggested the use of AFM as a tool to in vivo study explanted cells, providing an accurate measurement of stiffness or roughness variation associated to pathological state of cells, in comparison with healthy ones from the same patient [75]. Indeed, cell stiffness may be used as an indicator of different functionalities of cells in the presence of infections, inflammation or diseases of health or pathological tissues, [76] while surface roughness of cell membrane may be considered as a not invasive biomarker of cell surface [77]. For example, AFM has been used to detect early stages of cartilage degeneration in the case of osteoarthritis, with a precision not comparable with the most common available techniques [78]. Most interestingly, AFM may represent a powerful tool for the characterization of chemically modified surfaces of biomaterials, generally used in regenerative medicine and implants. Measurements of mechanical parameters such as elastic modulus of an implants interface may be extremely relevant to investigate the ability to in vivo resist to the attack of enzymes or other micro environmental molecules, thus giving a relevant contribution to increase the durability of implanted devices in the human body [79]. For instance, a recent modification of AFM tool involving adhesion measurement—namely Force Spectroscopy—has been adopted to calculate bond energies associated to local interactions between adjacent molecules, i.e., intermolecular forces or between different parts of single molecules, i.e., intramolecular forces [80]. By modifying AFM tips, it is possible to perform a complete characterization of interaction forces (i.e., colloidal, hydrophobic, hydrophilic and chiral forces) in biological phenomena [81] such as protein folding and unfolding [82,83,84,85] or DNA mechanics. Moreover, AFM tip can be functionalized with chemical groups, biomolecules, proteins and viruses, as well as with isolated living cells, to measure local forces across the sample, thus providing a direct access to several inter- and intramolecular and cellular phenomena, instead not detectable by other techniques. 

The investigation of interaction forces among ligands and receptors and their dynamics has been firstly overviewed by Hinterdorfer and Dufrene [86]. Since 1994, intra and intermolecular forces of the DNA have been measured via AFM by Lee et al. [87], providing a quantitative measurement of force to break DNA double helix conformation. They clearly verify analytical capacity to identify relative position of specific base sequences at higher resolution (i.e., angstrom level). Similarly, Rief et al., have measured mechanical properties depending upon the DNA sequence by stretching individual double strands connecting gold surface to the AFM tip [88]. Yu et al., in 2013 [89] used AFM force spectroscopy to characterize the adhesive force of four different asphalt binders, recognizing two micron-sized domains and nanometric height by topographic images, as a function of their different chemical and mechanical properties (i.e., sticky polymeric materials [90]). 

Moreover, single cell force spectroscopy may be successfully used for evaluating cell attachment onto biomaterials and medical devices. In this approach, cells are used as a force probe: the living cells are directly attached at the end of the AFM cantilever and probed towards the substrate. By using single cell force spectroscopy, Bertoncini et al. measured the adhesion properties of living mesenchymal stem cell (MSCs) on different type of titanium dioxide substrate [91], converting cell interactions into force probe as firstly reported by Taubenberger [92].

Noteworthy, higher benefits of using single-molecule force spectroscopy mainly concerns their applications in drug-delivery, as for nanostructured biomaterials for targeted or delayed release of antibiotics, growth factors and viruses. Indeed, strength of the specific interactions among bioactive molecules certainly influences the tendency to desorption, ultimately influencing ultimate release profile. Alternatively, single cells can be handily fixed onto the cantilever, working as probe for quantification of cell materials interactions forces [93].

Hence, force spectroscopy may be successfully used for dipping the knowledge of numerous processes involving porous scaffolds, bioactive molecules and cells, not merely limited to the optimization of biomaterials properties for tissue engineering use, but extending their potential to drug delivery and clinical applications, to define predictive models to quantify structures and interactions at the molecular level for the understanding of basic mechanisms of unknown diseases or the discovery of alternative pharmacological therapeutic treatments.

## 4. Advanced Tools 

### 4.1. Conductive AFM

**Figure 5:**
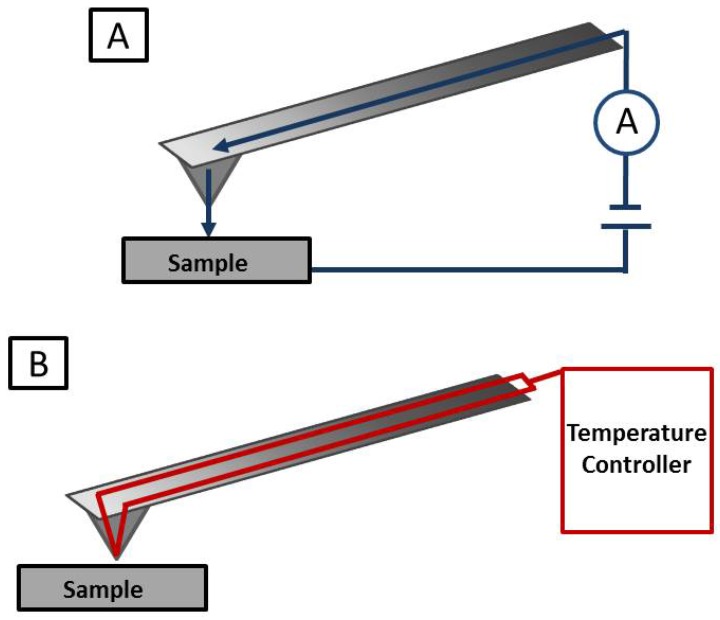
Schematic view of (**A**) Conductive AFM and (**B**) Thermal-AFM.

Conductive atomic force microscopy (C-AFM) is a robust technique also for electrical investigations in small-scale technologies, which could help scientists to explore the electrical properties and to reveal local conductivity of biomedical scaffolds made of conductive polymers. C-AFM employed the use of a nanometre-scale electrical probe that allows to simultaneously measuring the morphology and the current dispersion above the sample surface. It is well known, in fact, that among different materials, conducting polymers can be used to simplify the interaction between the neural system and the scaffold for regenerative purposes. The capability of conductive materials to regulate, induce and promote cell adhesion and development, seem to be very interesting for the tissue engineering community. Specifically, the use of conductive atomic force microscope is very advantageous in order to explore the nanoscale conductivity of conductive polymeric scaffold under stress. In C-AFM a bias voltage, accrued from the tip, is discharged on the sample and the tunnelling current among them is monitored (Figure 5A). Here the contact mode is used to produce a topography map while the conductive cantilever scans the surface for measuring the electrical signal, from the same sample area. The electrical signal is detected by an electric current amplifier, while the morphology is captured by monitoring the deflection of the cantilever [93]. The most important feature in this AFM advanced tool is the conductive tip. The resistant to corrosion and the good conductivity of the silicon probe coated with diamond thin layer has been proved several times. Therefore, these are the most suitable probe for the C-AFM. Beside the possibility to acquire simultaneously the conductivity and the topographic maps of the same area of a sample, this advanced tool has been used to investigate differences in the conductivity and in piezoelectricity for several polycrystalline thin films [94]. Moreover, in recent years, among different biomedical materials, the advantage related to the use of conductive polymers suggests a promising and encouraging future for them in all the medical fields. Due to the cytocompatibility, and good conductivity, conductive polymers, in the past, have been broadly studied for both biological and medical applications. It is well known that these polymers are able to exhibit excellent electrical properties that can be used to investigate the neuronal induction and/or the cardiac activity. The capability of electrical conductibility makes these polymers very well suitable for investigate the electrical behaviours of biological tissues.

The C-AFM as a tool able to simultaneously characterize conductivity and topography of polymeric films has been extensively used in the last decade [95,96]. For example, Pelto et al. have investigated the conductivity of polypyrrole-hyaluronic acid thin films in order to enhance stem cell response for tissue engineering scaffolds [97].

Finally, Lee and co-workers [98] have studied the conductivity of polyvinylidene difluoride (PVDF) microstructures under predefined stress via C-AFM. In their work, the same region of the sample was scanned before and after the application of an electrical signal. The results showed that under the application of an electrical signal, the surface profile encountered a morphological change bringing an increase in roughness. They found that electrical potential can influence surface morphology and microstructure of PVDF sample. 

### 4.2. Thermal Analyses

Another important feature of AFM concerns its attitude to be combined with thermal analysis for a more fine characterization of thermal properties of different regions of heterogeneous samples. This innovative AFM-related tool also recognized as nano-thermal analysis (nTA) allows for a local thermometric analysis [99]. Main advantage to assess thermal analyses at nanoscale, concerns the identification of changes in the polymer crystal state, also in the case mechanical properties do not change. As for the C-AFM, this is accomplished by using a specific tip at the end of an AFM probe. The cantilever is heated during the measurements, while the probe is pushed toward the sample until its volumetric expansion, due to a local heating from the cantilever (Figure 5B). The swelling of the sample pushes the probe up and causes an increase of the cantilever vertical bending. This deflection is, thus, evaluated using the photodetector system of a standard AFM. Due to the transition temperature, the sample suffer a change in the internal temperature, becoming softer than the initial state; as a consequences, the cantilever bending decreases and the probe is pushed in the sample causing elastic or plastic deformation [99]. By monitoring the cantilever deflection, the material thermal transition can be determined. Moreover, with this technique, the transition temperature of a sample can be positively correlated with the bulk measurements of the transition temperature obtainable with macroscopic thermal approach (i.e., thermomechanical and calorimetric analysis). In contrast with the bulk techniques, the nTA allows to investigate local thermal properties of a sample at micro and nano scale. This technique, therefore, can be definitely used to identify thermal properties of specific polymers and/or biomaterials. The cantilever is usually made of silicon and with different dopant concentrations, while the probe is a standard etched silicon probe that allows high spatial resolution analysis in both contact and tapping mode. The cantilever in this configuration can achieve very high temperatures, since the silicon doping allows it to work with high currents. Therefore, it is possible to claim that this technique is ideal for the chemical characterization of polymers in view of the wide range of temperatures that can be employed [100]. The use of thermal probe at the end of an AFM cantilever to study the denaturation of collagen fibrils has been reported by Bozec and Odlyha [101]. Their experiments have shown as both collagen and Gelatine samples underwent a two stage degradation transitions at higher temperature. In particular, the temperature was similar for both samples in the first degradation phase and significantly higher in the case of Gelatine for the second degradation transition. This study proves how useful can be the implications of thermal AFM in the biophysics and tissue engineering fields.

## 5. Conclusions and Future Trends

Atomic force microscopy (AFM) is becoming one of the most meaningful techniques among the scanning probe microscopes (SPM) for the investigation of surface forces at the nanometre scale and the imaging of micro-nanostructured surfaces. In the case of soft matter, i.e., polymers for biomedical use–AFM may assure an accurate scan of surface morphology, thus providing a strict correlation between morphological changes and process conditions [102]. Moreover, it can be used for characterizing nanocomposite materials in terms of mechanical, physical, thermal and chemical properties by approaching surface by different advanced tools. By means of phase image AFM, for instance, it is possible to detect elasticity and sample viscosities in the case of different substrates, from living cells to porous scaffolds until natural tissues such as bone and cartilage. More interestingly, adhesion maps may be generated collecting detailed information about heterogeneities on sample surfaces at nanometre resolution. Finally, the ability to distinguish polymer phase transition may support the understanding of peculiar properties in the case of natural or synthetic polymers. In other terms AFM is suitable to perform a complete and detailed characterization of scaffolds for tissue engineering applications. 

In this perspective, all the described approaches could be further implemented with recent discoveries on the use of AFM applied to biological systems able to achieve measurements of inter- and intramolecular interaction forces at the pico-newton resolution, in order to investigate specific interactions among biological molecules, biomaterials and natural tissues. To date, this approach based on the biological modifications of nanostructures is just successfully applied in different research areas, from nanomedicine, to biosensing and nanoelectronics, [103]. Mechanical, optical, and electric properties of nanoscale structures are mainly governed by quantum mechanics and molecular conjugation to control nanoparticles assembly, modulating their properties and tagging them for specific recognition or detection. In this context, AFM may be efficiently used to monitor the dynamics of bioconjugate species without any labelling modification in physiological solution at high temporal (~100 ms) and sub-molecular spatial resolution [104,105]. As a function of AFM sensitivity, this will assure the detection of specific interaction forces and the manipulation of single molecules, by deeply understanding the peculiar mechanisms intrinsically involved, thus paving the way towards their use as promising analytic and diagnostic tool for the near future. Furthermore, a combinatorial approach based on an integrated use of AFM with optical microscopies may be useful to detect and recognize ligands and receptors through bioconjugated AFM tips [106], or to manipulate single nanostructures [107], further expanding the range of simultaneously accessible information in terms of structural and functional properties of nanoparticles/nanofibres. Alternatively, AFM probing techniques may be adapted to perform measurement of liquid viscosity, through the assessment of torsion in AFM cantilever as a whisker tip scanning occurs inside liquid medium [108]. These techniques are recently becoming indispensable for assessing the effect of blood-thinning drugs like aspirin, coumarin, heparin, dipyridamole, clopidogrel, etc. and for the development of novel therapeutic agents with potential effect on blood viscosity [109]. Besides, AFM may be used not only to collect and analyse the properties of nanomaterials, but also to fabricate ex novo custom-made structures by the use of nanorobotics tips able to work as sharp stylus to scratch a substrate surface forming nanopatterns in a nanolithography-type approach [110]. AFM-assisted technologies such as nanooxidation, force lithography, nanografting, and dip-pen nanolithography—with tips radius ranging from several nanometres to tens of nanometres—may be just used to modify the sample surface, either physically or chemically, at nanometre scale [111] for nanopatterning semiconductors, metals, polymers, etc., in nanoelectronics, bioanalysis, biosensors, actuators and high-density data storage devices [112]. In perspective, this approach will be progressively addressed to life sciences, opening the route towards “molecular writing” of surfaces where molecules could be delivered from the AFM tip via capillary transport as a function of the peculiar chemical affinity with solid-state substrate.

## Figures and Tables

**Table 1:** Summary of AFM modes of operation.

AFM Modes of Operation	Working Principle	Advantage	Disadvantage
Contact Mode	Physical contact between the tip and the surface	High scan speeds High resolution	Damage to soft sample Later forces may produce image artefacts
Non-contact Mode	No contact between the tip and the sample	Low resolution No damage to sample	Slower scan speed if compared with both contact and tapping mode
Tapping Mode	Intermittent and short contact between the sample and the tip	High resolution Minimal damage to sample	Slower scan speed if compared with contact mode
